# Effectiveness and knowledge, attitudes and practices of seasonal influenza vaccine in primary healthcare settings in South Africa, 2010–2013

**DOI:** 10.1111/irv.12305

**Published:** 2015-04-23

**Authors:** Johanna M McAnerney, Sibongile Walaza, Adam L Cohen, Stefano Tempia, Amelia Buys, Marietjie Venter, Lucille Blumberg, Jazmin Duque, Cheryl Cohen

**Affiliations:** a National Institute for Communicable Diseases (NICD) of the National Health Laboratory Services (NHLS)Johannesburg, South Africa; b U.S. Centers for Disease Control and PreventionAtlanta, GA, USA; c U.S. Centers for Disease Control and PreventionPretoria, South Africa; d Battelle AtlantaAtlanta, GA, USA

**Keywords:** attitudes, Influenza-like illness, knowledge, practices, vaccine

## Abstract

**Objectives:**

Influenza vaccine effectiveness (VE) and coverage data for sub-Saharan Africa are scarce. Using a test-negative case–control design, we estimated influenza VE annually among individuals with influenza-like illness presenting to an outpatient sentinel surveillance programme in South Africa from 2010 to 2013. A knowledge, attitudes and practices (KAP) influenza vaccine survey of programme clinicians was conducted in 2013.

**Sample:**

In total, 9420 patients were enrolled in surveillance of whom 5344 (56.7%) were included in the VE analysis: 2678 (50.1%) were classified as controls (influenza test-negative) and 2666 (49.9%) as cases (influenza test-positive).

**Results:**

Mean annual influenza vaccine coverage among controls was 4.5% for the four years. Annual VE estimates adjusted for age, underlying medical conditions and seasonality for 2010-2013 were 54.2% (95% confidence interval (CI): 2.4–78.6%), 57.1% (95% CI: 15.5–78.2%), 38.4% (95% CI: −71.7–78.1%) and 87.2% (95% CI: 67.2–95.0%), respectively. The KAP survey showed that >90% of clinicians were familiar with the indications for and the benefits of influenza vaccination.

**Conclusions:**

Our study showed that the vaccine was significantly protective in 2010, 2011 and 2013, but not in 2012 when the circulating A(H3N2) strain showed genetic drift. Vaccine coverage was low despite good clinician knowledge of vaccination indications. Further studies are needed to investigate the reason for the low uptake of influenza vaccine.

## Introduction

Influenza vaccine has been available since the mid-1940s and remains the most effective method to prevent influenza disease. Vaccine must be given annually because of potential drift of strains causing disease each year.^1^ Annual estimates of influenza vaccine effectiveness (VE) are of public health benefit and may help demonstrate whether the vaccine works in a given year and setting. Little is known about influenza VE in sub-Saharan Africa and the barriers to vaccine delivery. Southern Hemisphere trivalent inactivated influenza vaccine is the only influenza vaccine available in South Africa and has been available in the private sector for many years. Guidelines for the use of influenza vaccine are published annually in South Africa.^2^ Since 2009, a limited number of doses have been made available in the public sector for high-risk groups, which include young children, the elderly, pregnant or post-partum (within 2 weeks of delivery) women, and persons of any age with underlying medical conditions (such as heart disease, lung disease and HIV infection). In addition, in the private sector, any person wishing to protect themselves from the risk of contracting influenza is eligible to receive vaccine. In 2010, the South African Department of Health initiated the first national influenza vaccination campaign, held annually before the influenza season from March to May^3^. Uptake of influenza vaccine remains low in South Africa. Fewer than 1 million doses have been distributed in the public and private sectors over the past 3 years, despite estimates that more than 20 million South Africans are in groups at high risk of severe influenza disease.^3^ The number of doses of influenza vaccine imported into South Africa is based on the uptake of the vaccine during the previous year. Low uptake of vaccine was not thought to be due to shortage of vaccine.

Sentinel influenza surveillance systems can provide platforms for the estimation of influenza VE. Several such surveillance systems have been used to annually estimate VE, although none from Africa.^4^–^6^ South Africa has long-standing sentinel surveillance through the Viral Watch Programme (VWP), a network of primary healthcare providers predominantly in the private health sector, which was started in 1984 to describe influenza seasonality in South Africa and to provide influenza strains for global vaccine strain selection.^7^ We estimated annual influenza VE from 2010 to 2013 in primary healthcare settings in South Africa, and we assessed knowledge, attitudes and practices (KAP) of influenza vaccine among VWP practitioners.

## Methods

### Influenza surveillance through the VWP

As described previously, the VWP is a national sentinel influenza surveillance programme consisting of approximately 180 outpatient practitioners, of whom 90% are general practitioners of all race groups in all socioeconomic areas; however, only 11 (6%) were based at clinics in the public sector.^7^ Of the nine provinces of South Africa, the largest percentage of participating VWP practitioners during 2010–2013 were in Gauteng (40–47% of practitioners), which has 23% of the population of South Africa, and the Western Cape (23–28% of practitioners), which has 11% of the population. Influenza-like illness (ILI) was defined as an acute respiratory illness with a measured temperature of ≥38°C or a history of fever, and cough, with onset within the past 7 days. Clinical, demographic and influenza vaccination data were collected from each patient at the time of nasopharyngeal swab collection.^7^ Data on race and socioeconomic group were not collected. Practitioners were supplied with VW specimen forms to record underlying medical conditions using a tickbox format. Details were completed from medical records where available, or by self-report. Practitioners were limited to submitting no more than five specimens per week throughout the year to avoid overburdening the laboratory, and the choice of patient was left to the individual practitioner's discretion. Specimens were tested using multiplex reverse transcription real-time polymerase chain reaction (rRT-PCR) assays for influenza A and B as well as other respiratory viruses. Influenza-A-positive specimens were further subtyped by rRT-PCR.^8^ Specimens from seven of the nine provinces were tested at the National Institute for Communicable Diseases (NICD), whereas specimens from the other two provinces were tested at central laboratories in those provinces using the same test and assay. All positive specimens were sent to the NICD for sequencing.

### Estimating VE

A test-negative case–control study was conducted among patients enrolled as part of the VWP to estimate the VE of the Southern Hemisphere TIV. Patients from all nine provinces presenting with ILI to participating centres of the VWP from 1 January 2010 to 31 December 2013 were eligible for inclusion. Patients who met the ILI case definition, had a known influenza vaccine history and were 6 months or older were included in the VE analysis. Vaccine history was recorded from medical records where available, and if not was self-reported. It was not recorded whether children under 9 years had received two doses. Patients were considered to be vaccinated if they had received influenza vaccine >14 days prior to the onset of illness. Patients who had received vaccine ≤14 days prior to onset of symptoms were excluded from the study. Patients in whom influenza was detected were considered cases and those who tested negative for influenza were unmatched controls.^9^–^12^ Patients who presented at a clinic at the international arrivals of the major airport into South Africa were excluded as it was assumed that these patients contracted influenza outside of South Africa.

The start of the influenza season was defined as the week when at least two consecutive weekly influenza detection rates of ≥10% in specimens received from the VWP, and the season was considered to have ended when the detection rate dropped below 10% for two consecutive weeks, or when the number of specimens received were <10 per week. Only specimens collected during the season were included in the VE analysis. Each influenza season was divided into three equal parts, that is early, mid and late season, to assess the possibility of waning immunity during the season.^12^,^13^ As there were too few patients in the extremes of age, three age groups were selected for analysis, that is children and teenagers (<20 years), young adults (20–44 years) and older adults (≥45 years).

The Southern Hemisphere influenza vaccine for 2010–2012 was unchanged and contained an A/California/7/2009 (H1N1)-like virus, an A/Perth/16/2009 (H3N2)-like virus, and a B/Brisbane/60/2008-like virus. For 2013, the vaccine contained an A/California/7/2009 (H1N1)-like virus, an A/Victoria/361/2011 (H3N2)-like virus, and a B/Wisconsin/1/2010-like virus.

Categorical variables were compared using the chi-squared test, and multivariable logistic regression was used for the VE estimates to adjust for age, underlying medical conditions and season period where possible as we were not powered to obtain stratum-specific estimates. VE was calculated as 1 odds ratio (OR) for laboratory-confirmed influenza in vaccinated and unvaccinated patients.

Over the study period, there were approximately 1300 patients eligible (according to the above-mentioned criteria) for VE calculations on average every year, with a mean annual influenza detection rate of about 50% and a mean average vaccine coverage of about 5% among controls. Given the available data, we estimated that we would be able to obtain statistically significant VE estimates of approximately 40% and above assuming 95% confidence intervals and 80% power.

### KAP survey

To assess reasons for low vaccine uptake, a written survey on the knowledge, attitudes and practices regarding influenza vaccination was conducted among VWP practitioners during an annual Influenza Symposium in March 2013. In May 2013, the survey was sent out electronically requesting those who were unable to attend to complete the online survey. The survey consisted of 20 multichoice questions and one open-ended question. There were eight questions that assessed the practitioners' knowledge of influenza and influenza vaccine. The survey was anonymous with no demographic data required about the respondents.

### Ethical review

Ethical approval for this programme and survey was obtained from the University of the Witwatersrand Human Research Ethics Committee (Medical). Individual informed consent was not required as the test was performed for diagnostic purposes as part of surveillance. The Centers for Disease Control and Prevention's Institutional Review Board relied on the ethical approval from the University of the Witwatersrand.

## Results

### Vaccine coverage and VE analysis

**Figure 1:**
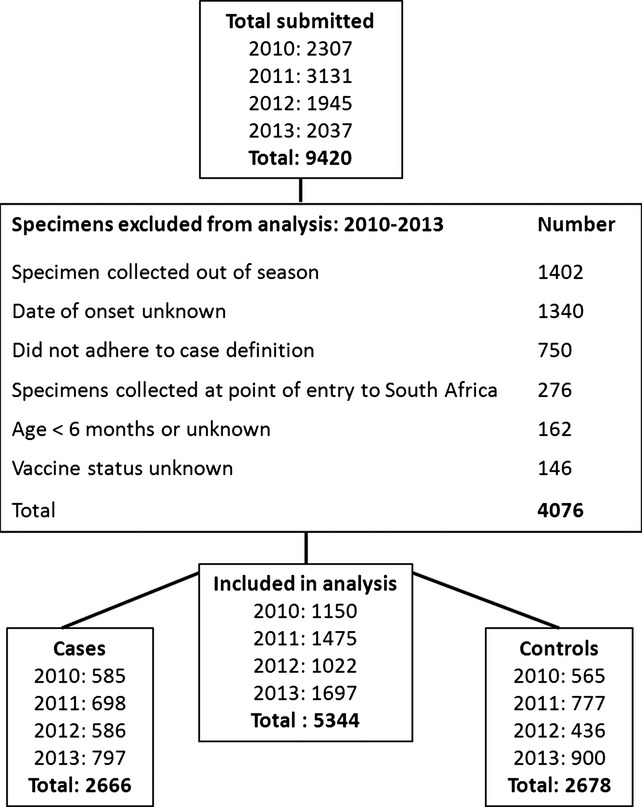
Flow of specimens and allocation of cases and controls for vaccine effectiveness analysis – South Africa, 2010–2013.

In total, 9420 patients with ILI were enrolled over the 4-year study period (Figure1). Of these, 5344 (56·7%) were included in the VE analysis. Of the 4076 excluded specimens, 1402 (34·4%) were collected out of season, 1340 (32·9%) had an unknown date of illness onset, 750 (18·4%) did not meet the ILI case definition, 276 (6·8%) were collected at the point of entry into South Africa, 162 (4·0%) were less than 6 month of age or age unknown, and 146 (3·6%) had unknown influenza vaccine status. Among included individuals, 2666 (49·9%) were classified as cases (influenza test-positive) and 2678 (50·1%) as controls (influenza test-negative).

During the 4-year period, the annual influenza season occurred between May and September. The dominant types or subtypes differed by year with influenza B accounting for 310/585 (53·0%) of total influenza-positive cases in 2010, influenza A(H1N1)pdm09 for 561/698 (80·4%) in 2011, influenza A(H3N2) for 360/586 (61·4%) in 2012 and influenza A(H1N1)pdm09 for 539/797 (68·0%) in 2013 (Table1).

**Table 1:** Circulating influenza strains, percentage of total influenza-positive cases and vaccine strains per year: Viral Watch Programme 2010–2013

Year	Circulating strains	Percentage of total influenza-positive cases	Vaccine strains
2010	A/California/7/2009 (H1N1)	20	A/California/7/2009 (H1N1)
	A/Perth/16/2009 (H3N2)	27	A/Perth/16/2009 (H3N2)
	B/Brisbane/60/2008	53	B/Brisbane/60/2008
2011	A/California/7/2009 (H1N1)	80	A/California/7/2009 (H1N1)
	A/Perth/16/2009 (H3N2)	12	A/Perth/16/2009 (H3N2)
	B/Brisbane/60/2008	8	B/Brisbane/60/2008
2012			A/California/7/2009 (H1N1)
	A/Victoria/361/2011 (H3N2)	61	A/Perth/16/2009 (H3N2)
	B/Brisbane/60/2008B/Florida/4/2006	39	B/Brisbane/60/2008
2013	A/California/7/2009 (H1N1)	68	A/California/7/2009 (H1N1)
	A/Victoria/361/2011 (H3N2)	17	A/Victoria/361/2011 (H3N2)
	B/Wisconsin/1/2010	15	B/Wisconsin/1/2010

Nearly half (2569, 48·1%) of specimens came from patients aged 20–44 years, 2564 (48·0%) of patients were male, and 2951 (55·2%) of patients attended VWP sites in the central plateau region with a temperate climate (Free State, Gauteng, Northern Cape and North West provinces). A total of 4573 (85·6%) specimens were collected during the early (first third) and mid (second third)part of the influenza season. The majority of specimens 4913 (91·9%) were collected within 3 days of onset of symptoms. Six hundred and seventy-seven (12·7%) patients had an underlying medical condition, such as chronic pulmonary and cardiac disease (320; 47·3%), HIV infection (96; 14·3%), other immunosuppression (82; 12·0%), metabolic disorders (121; 17·9%), pregnancy and post-parturition (55; 8·1%), and morbid obesity defined as a body mass index of ≥40 (3; <1%). Depending on year, there were differences in age, seasonality, region and interval between onset and sampling between cases and controls (Table2).

**Table 2:** Characteristics of cases (influenza test-positive) and controls (influenza test-negative) in the Viral Watch Programme, South Africa, 2010–2013

Variable	2010		2011		2012		2013	
	Cases (*N* = 585) *n* (%)	Controls (*N* = 565) *n* (%)	Cases (*N* = 698) *n* (%)	Controls (*N* = 777) *n* (%)	Cases (*N* = 586) *n* (%)	Controls (*N* = 436) *n* (%)	Cases (*N* = 797) *n* (%)	Controls (*N* = 900) *n* (%)
Age group	*P* < 0·001		*P* < 0·001		*P* = 0·200		*P* = 0·415	
6 months–19 years	248 (42·4)	150 (26·5)	213 (30·5)	206 (26·5)	195 (33·2)	135 (31·0)	235 (29·5)	255 (28·3)
20–44 years	246 (42·1)	302 (53·5)	371 (53·2)	378 (48·6)	250 (42·7)	210 (48·2)	388 (48·7)	424 (47·1)
≥45 years	91 (15·6)	113 (20·0)	113 (16·2)	193 (24·8)	141 (24·1)	91 (20·9)	174 (21·8)	221 (24·6)
Sex	*P* = 0·613		*P* = 0·250		*P* = 0·148		*P* = 0·242	
Male	307 (52·5)	282 (49·9)	331 (47·4)	335 (43·1)	309 (52·7)	205 (47·0)	392 (49·2)	408 (45·3)
Female	275 (47·0)	281 (49·7)	366 (52·4)	441 (56·8)	276 (47·0)	229 (52·5)	401 (50·3)	489 (54·3)
Unknown	3 (0·5)	2 (0·4)	1 (0·1)	1 (0·1)	2 (0·3)	2 (0·5)	4 (0·5)	3 (0·3)
Seasonality*	*P* < 0·001		*P* < 0·001		*P* = 0·049		*P* < 0·001	
Early	134 (22·9)	205 (36·3)	506 (72·5)	461 (59·3)	174 (29·7)	150 (34·4)	349 (43·8)	305 (33·9)
Mid	383 (65·5)	288 (51·0)	135 (19·3)	255 (32·8)	294 (50·2)	185 (42·4)	315 (39·5)	434 (48·2)
Late	68 (11·6)	72 (12·7)	57 (8·2)	61 (7·9)	118 (20·1)	101 (23·2)	133 (16·7)	161 (17·9)
Region**	*P* < 0·001		*P* < 0·001		*P* = 0·343		*P* < 0·001	
Central plateau	462 (79·0)	343 (60·7)	428 (61·3)	425 (54·7)	288 (49·1)	195 (44·7)	413 (51·8)	400 (44·4)
North-east subtropical	72 (12·3)	87 (15·4)	158 (22·6)	151 (19·4)	89 (15·2)	76 (17·4)	196 (24·6)	258 (28·7)
Southern coastal belt	51 (8·7)	135 (23·9)	112 (16·0)	201 (25·9)	209 (35·7)	165 (37·8)	188 (23·6)	242 (26·9)
Underlying medical condition***	*P* = 0·225		*P* = 0·332		*P* = 0·401		*P* = 0·676	
None	475 (81·2)	467 (82·7)	526 (75·4)	604 (77·7)	427 (72·9)	319 (73·2)	630 (79·0)	711 (79·0)
Yes	54 (9·2)	59 (10·4)	87 (12·5)	97 (12·5)	81 (13·8)	50 (11·5)	122 (15·3)	130 (14·4)
Unknown	56 (9·6)	39 (6·9)	85 (12·2)	76 (9·8)	78 (13·3)	67 (15·4)	45 (5·6)	59 (6·6)
Interval between onset and sampling	*P* = 0·260		*P* = 0·005		*P* = 0·251		*P* = 0·002	
0–3 days	535 (91·5)	494 (87·4)	663 (95·0)	709 (91·2)	551 (94·0)	402 (92·2)	750 (94·1)	809 (89·9)
4–7 days	50 (8·5)	71 (12·6)	35 (5·0)	68 (8·8)	35 (6·0)	34 (7·8)	47 (6·0)	91 (10·1)

* Early = first third of influenza season, Mid = middle third of influenza season, Late = last third of influenza season.

** Central plateau = Free State, Gauteng, Northern Cape and North West provinces; north-east subtropical = KwaZulu-Natal, Mpumalanga and Limpopo provinces; southern coastal belt = Eastern Cape and Western Cape Provinces.

*** Chronic pulmonary and cardiac disease, chronic renal disease, diabetes and similar metabolic disorders, immunosuppression, morbid obesity (BMI ≥ 40), and pregnancy and post-parturition.

The *P* value calculated using Pearson's chi-squared test refers to the difference between cases and controls.

Annual influenza vaccine coverage in patients included in the VE analysis was low (a mean of 1·4% and 4·5% for cases and controls, respectively; Table3). Coverage was highest in those ≥45 years of age (2·9% and 7·0% for cases and controls, respectively) and lowest in those <20 years of age (1·2% and 2·1% for cases and controls, respectively; Table3). Over the 4-year period, 116/158 (73·4%) of patients received their vaccine before the onset of the season and an additional 36 (22·8%) received it before the peak of the season. Vaccinated cases had received influenza vaccine between 3 weeks and 6 months prior to onset of symptoms. Influenza vaccine was found to be effective in three of the 4 years (Table3). VE estimates adjusted for age, underlying medical conditions and seasonality for 2010–2013 were 54·2% (95% CI: 2·4–78·6%) for 2010, 57·1% (95% CI: 15·5–78·2%) for 2011, 38·4% (95% CI: −71·7–78·1%) for 2012 and 87·2% (95% CI: 67·2–95·0%) for 2013.

**Table 3:** Vaccine coverage and vaccine effectiveness (VE) estimates by year, underlying medical condition (UMC), age group and timing within season, Viral Watch Programme, South Africa, 2010–2013

Year	Subgroup	Vaccine coverage			Percentage Unadjusted VE (95% CI)
		Total *n*/*N* (%)	Cases *n*/*N* (%)	Controls *n*/*N* (%)	
2010	Total	41/1150 (3·6)	11/585 (1·9)	30/565 (5·3)	48·2 (13·8, 68·8)
	UMC*	7/113 (6·2)	1/54 (1·9)	6/59 (10·2)	71·4 (−77·1, 95·4)
	<20 years	6/398 (1·5)	3/248 (1·2)	3/150 (2·0)	20·0 (−78·7, 64·2)
	20–44 years	17/548 (3·1)	4/246 (1·6)	13/302 (4·3)	48·4 (−22·2, 78·2)
	≥45 years	18/204 (8·8)	4/91 (4·4)	14/113 (12·4)	52·5 (−14·3, 80·3)
	Early season	20/339 (5·9)	4/134 (3·0)	16/205 (7·8)	50·9 (−19·1, 79·8)
	Mid-season	17/671 (2·7)	6/383 (1·6)	11/288 (3·8)	38·8 (−16·9, 67·9)
	Late season	4/140 (2·9)	1/68 (1·5)	3/72 (4·2)	49·3 (−179·4, 90·8)
2011	Total	52/1475 (3·5)	14/698 (2·0)	38/777 (4·9)	44·0 (12·1, 64·3)
	UMC*	11/184 (6·0)	4/87 (4·6)	7/97 (7·2)	24·2 (−68·2, 65·8)
	<20 years	8/420 (1·9)	5/214 (2·3)	3/206 (1·5)	−23·2 (−112·5, 28·6)
	20–44 years	24/749 (3·2)	5/371 (1·3)	19/378 (5·0)	58·7 (9·7, 81·1)
	≥45 years	20/306 (6·5)	4/113 (3·5)	116/193 (8·3)	47·5 (−27·6, 78·4)
	Early season	26/967 (2·7)	9/506 (1·8)	17/461 (3·7)	34·5 (−11·5, 64·9)
	Mid-season	22/390 (5·6)	3/135 (2·2)	19/255 (7·5)	62·0 (−9·8, 86·8)
	Late season	4/118 (3·4)	2/57 (3·5)	2/61 (3·3)	−3·6 (−181·2, 61·8)
2012	Total	18/1022 (1·8)	8/586 (1·4)	10/436 (2·3)	22·8 (−29·7, 54·1)
	UMC*	4/131 (3·1)	1/81 (1·2)	3/50 (6·0)	60·3 (−117·8, 92·8)
	<20 years	3/330 (0·9)	2/195 (1·0)	1/135 (0·7)	−13·0 (−152·7, 49·5)
	20–44 years	11/460 (2·4)	2/250 (0·8)	9/210 (4·3)	67·1 (−15·6, 90·6)
	≥45 years	4/232 (1·7)	4/141 (2·8)	0/91 (0·0)	−66·4 (−85·0, −49·7)
	Early season	6/263 (2·3)	2/138 (1·4)	4/125 (3·2)	37·0 (−96·4, 79·8)
	Mid-season	11/612 (1·8)	5/384 (1·3)	6/239 (2·5)	26·6 (40·6, 61·7)
	Late season	1/136 (0·7)	1/64 (1·6)	0/72	−114·3 (−156·6, −78·9)
2013	Total	47/1697 (2·8)	5/797 (0·6)	42/900 (4·7)	77·8 (49·0, 90·3)
	UMC*	12/252 (4·8)	2/122 (1·6)	10/130 (7·7)	66·3 (−20·4, 90·5)
	<20 years	9/490 (1·8)	1/235 (0·4)	8/255 (3·1)	77·1 (−45·9, 96·4)
	20–44 years	22/812 (2·7)	1/388 (0·3)	21/424 (5·0)	90·7 (36·8, 98·6)
	≥45 years	16/395 (4·1)	3/174 (1·7)	13/221 (5·9)	58·3 (−16·3, 85·1)
	Early season	20/654 (3·1)	3/349 (0·9)	17/305 (5·6)	72·5 (21·8, 90·3)
	Mid-season	22/749 (2·9)	2/315 (0·6)	20/434 (4·6)	78·9 (20·6, 94·4)
	Late season	5/294 (1·7)	0/133 (0·0)	5/161 (3·1)	Unable to calculate

* UMC, underlying medical conditions.

### KAP survey

Of the 167 registered VWP practitioners in 2013, 14 could not be reached. The survey was completed by 96/153 (62·7%) of practitioners (11 at the Influenza Symposium and the remainder electronically). The majority of VWP members to whom the KAP survey was administered were general practitioners 154/167 (92·2%), nine (5·4%) were occupational health nurses or doctors, and 4 (2·4%) were paediatricians.

The vast majority (94/96, 97·9%) of respondents agreed that it was possible for patients to be hospitalised or die from influenza. More than 85% of the 96 respondents correctly identified pregnant women, persons older than 65 years, young children, persons with human immunodeficiency virus (HIV)/acquired immunodeficiency syndrome (AIDS), persons with asthma or other chronic lung conditions, persons with tuberculosis and persons with chronic medical conditions as groups more likely to have influenza-related complications such as pneumonia or bronchitis. However, 43 (44·7%) of respondents felt that healthcare workers were also at higher risk of complications as well. Sixty-four (68·1%) of 94 respondents thought the vaccine to be highly effective, 29/94 (30·1%) thought it to be of average effectiveness, and one respondent did not think that influenza vaccine was effective.

Of the 96 participants, 85 (88·5%) responded that South Africa has a national recommendation regarding seasonal influenza vaccine and 86/92 (93·5%) knew that vaccine needed to be given annually. Of those who knew the vaccine needed to be given annually, 53/86 (61·6%) specified a time. Of these respondents, 32/53 (60·4%) answered that the vaccine should be given between March and July; 21 (39·6%) correctly responded more specifically that the vaccine should be given during autumn months (March–May) or before the influenza season. In addition, 82/96 (85·4%) agreed that vaccinating healthcare workers protected patients from becoming infected with influenza. Of the 96 respondents, 87 (90·6%) had previously been vaccinated against influenza and had received the vaccine in 2012. Seventy-seven (80·2%) had ready access to the vaccine, and 15 (15·6%) were able to get it free of charge. The three main reasons given for being vaccinated were to protect themselves and their patients against influenza (95, 99·0%), to protect family members against influenza (95, 99·0%) and to avoid missing work due to illness (88, 91·7%).

Although 83 (86·5%) practitioners mostly or always recommended influenza vaccine to their patients, 94 (97·9%) always recommended it to patients at risk of complications. The obstacles reported by practitioners to vaccinating patients were mainly patient refusal (59, 61·5%) and patients not coming to the practice when vaccinations were due (54, 56·3%). VWP practitioners also reported that there were several misconceptions regarding influenza vaccine influencing patient's decisions: that the vaccine caused influenza, that the vaccine had severe side effects and that influenza was always a mild illness.

## Discussion

This is the one of the first published articles describing influenza VE in sub-Saharan Africa. Estimates of VE ranged from 58 to 87% in three of the 4 years evaluated. These moderate VE estimates suggest a measurable benefit of vaccinating those at risk of severe influenza in our setting. Despite the fact that the majority of clinicians were familiar with the indications for and the benefits of influenza vaccination, coverage of vaccine was extremely low (∼2–3%) among patients with ILI attending practitioners participating in an influenza surveillance network. These favourable VE results should support healthcare practitioners to increase vaccine coverage in at-risk groups to prevent influenza.

The low VE point estimate in 2012 occurred when circulating A(H3N2) showed genetic drift that may have led to a vaccine mismatch. 14 VE estimates during the 2011–2012 season in Europe, which was also dominated by A(H3N2), were similar to our results.^11^–^13^,^15^,^16^ Both the 2011 and 2013 seasons in South Africa were dominated by A(H1N1)pdm09 with moderate–high VE point estimates; the 2013 value is similar to an Australian study which showed VE point estimates of 89% and 87% for 2009 and 2010 when A(H1N1)pdm09 circulated.^17^ This is expected as this strain has shown very little drift from the strain included in the vaccine. In New Zealand in 2013, when the season was dominated by A(H3N2), overall VE estimates were 56%.^18^

The low influenza vaccine coverage was similar to findings from a previous South African study which showed a mean vaccine coverage of 4% among VWP patients between 2005 and 2009.^19^ However, the VWP practitioners, many of whom have been part of the programme for many years and are well versed in the benefits of influenza vaccine, had a very high vaccine coverage of 90·6%. Although 86·5% of practitioners stated that they mostly or always offered influenza vaccine to their patients, the proportion of patients reached is not known. The annual vaccine is offered to patients attending the practice from when it becomes available in late summer. In a recent South African study of patients with lower respiratory tract infection, none of the >1000 influenza-positive patients had received influenza vaccine.^20^ Despite the vaccine being free of charge for at-risk groups, vaccine coverage of high-risk groups in the public sector has been reported to be as low as 2% among individuals aged >65 years, but up to 14% among pregnant women.^3^ This contrasts sharply with reported influenza vaccine coverage from the United States (45% in all children and adults attending a primary healthcare provider), Australia (59% in patients with medical comorbidities and 82% in patients aged ≥65 years) and Brazil (from 38% to 88% in a population >60 years of age).^21^–^23^ Some European countries, however, report lower influenza vaccine coverage of 3% in cases and 8% in controls from surveillance programmes similar to ours.^24^ Very little data are available on influenza vaccine coverage in other African countries.^25^ A study from Kenya reported the distribution of 30 000 doses of influenza vaccine for a population of approximately 30 million (0·001%).^26^ During the 2009 pandemic, the World Health Organization (WHO) distributed 32·2 million doses of pandemic vaccine to 32 countries in Africa with vaccine coverage ranging from 0·4% to 11% (median 4%).^25^

The KAP survey revealed that a commonly reported barrier to vaccination was patient refusal due to patient misconceptions. Such misconceptions included that the vaccine causes influenza, the vaccine causes severe vaccine side effects, and influenza is only a mild illness. Limited vaccine availability was not identified as a barrier to vaccination in the private healthcare sector. In a study in Côte d'Ivoire carried out during the 2009 pandemic, the main reasons for not being vaccinated were doubts about the efficacy of the vaccine and fear of adverse effects [254]. A similar study in Kenya reported that 89% of healthcare workers would accept the vaccine if it was available free of charge. However, very few healthcare worker respondents (4%) said they would get vaccinated to protect their patients.^26^ In contrast, 83% of practitioners who participated in our study stated that protecting their patients was a reason to be vaccinated. Despite the good knowledge of the benefits of influenza vaccine among the VWP practitioners, influenza coverage remains very low among their patients.

## Limitations

There are several limitations to our study. First, our study was not specifically designed to assess VE and, given the available data, we were not powered to statistically assess significance of VE estimates lower than 40%. Second, the low vaccine coverage affected the ability to statistically estimate significance of VE among smaller subgroups such as the elderly (>65 years of age). It is unlikely that those tested for influenza by the VWP practitioners are a random sample, and additional data on race and socioeconomic group were not available. As the number of specimens per practitioner per week was limited, a low proportion of patients would have been swabbed during periods of high influenza activity, and this may have biased our findings if included patients differed from those not swabbed. It was left to the discretion of the practitioner to choose the patients to be included for swabbing. Influenza vaccine status was self-reported by the patients to the practitioner which could have led to misclassification of vaccine status. These potential biases may have led to an over- or underestimation of vaccine effectiveness.

As only the current year's influenza vaccination history is recorded, we could not control for previous use of seasonal influenza vaccine, which has been suggested to diminish VE.^21^ Although we were able to stratify and adjust for intraseasonal changes, we did not have enough power to find intraseasonal waning of VE, which has been demonstrated in studies from Spain and the United Kingdom.^12^,^13^ According to the South African influenza vaccine recommendations, children under 9 years of age should receive two doses of influenza vaccine 1 month apart. During the 4-year period, only 14 children under the age of 9 years had received influenza vaccine (six cases, eight controls). However, it was not known whether they had received one or two doses. Lastly, patients were not interviewed; thus, the reasons for patient refusal of influenza vaccine were reported by the VWP practitioners. The results from the KAP survey are also not generalisable to other healthcare professionals.

## Conclusion

Our study showed that influenza vaccine was significantly protective in years 2010, 2011 and 2013, but not in 2012 when the circulating A(H3N2) strain showed genetic drift. Our VE estimates should encourage healthcare practitioners to vaccinate their patients, especially those at high risk of complications, against influenza on an annual basis. Providing healthcare providers with annual VE estimates as well as coverage rates for their practices should encourage them to increase influenza vaccine coverage in at-risk groups. Further studies among patients are needed to assess the reason for the low uptake of influenza vaccine.

## Authors' contributions

JMcA, SW, ALC, ST, JD and CC participated in conception and design of study; JMcA, ST, AB and MV participated in data collection and laboratory processing; JMcA, SW, ALC, ST, LB and CC participated in analysis and interpretation; and JMcA, SW, ALC, ST, AB, MV, LB, JD and CC participated in drafting or critical review of the article.
